# Highly Selective Hg (II) Ion Detection Based on Linear Blue-Shift of the Maximum Absorption Wavelength of Silver Nanoparticles

**DOI:** 10.1155/2012/856947

**Published:** 2012-04-03

**Authors:** Li Ping Wu, Hua Wen Zhao, Zhu Hong Qin, Xian Ying Zhao, Wen Dan Pu

**Affiliations:** ^1^Department of Chemistry, Third Military Medical University, Chongqing 400038, China; ^2^College of Chemistry and Chemical Engineering, MOE Key Laboratory on Luminescence and Real-Time Analysis, Southwest University, Chongqing 400715, China

## Abstract

A new method of detecting Hg (II) ion with silver nanoparticles (AgNPs) is developed in this contribution. When Hg (II) ions were added into AgNPs solution, the solution displayed rapid color change and blue shift of the maximum absorption wavelength (Δ*λ*), which was in proportion to the Hg (II) ion concentration over the range of 2.0 × 10^−7^–6.0 × 10^−6^ mol/L, with detection limit (3**σ**) of 6.6 × 10^−9 ^mol/L. Under the same experimental conditions, other metal ions did not interfere. Thus, we propose a rapid, simple and highly selective method for detecting Hg (II) ion.

## 1. Introduction

Hg (II), a widespread heavy metal, has been widely recognized as one of the most hazardous pollutants and highly carcinogenic materials due to its accumulative and toxic properties in the environment [1]. Accumulative properties of mercury may cause serious harm to human beings and animals. Moreover, Hg (II) can be transformed into methylmercury by bacteria [2], from which several serious disorders may arise, including sensory, mental, and neural damage [3]. Therefore, environmental monitoring of aqueous mercury becomes an increasing demand. By this time, methods of detecting mercury have been reported, such as colorimetric methods [4–7], atomic absorption spectrometry [8, 9], inductively coupled plasma mass spectrometry [10], mercury sensors with gold nanorods [11], and gold nanoparticles [12]. Although these methods have good sensitivity and selectivity, they are cost and time consuming. Therefore, it is necessary to develop a simple and rapid method.

Metal nanoparticles have been extensively studied and used because of their attractive optical, electronic, biological, and catalytic properties [13]. AgNPs have been paid more attention for special properties such as higher extinction coefficients, catalytically active and exhibiting Raman enhancement properties [14, 15]. Because of these properties, AgNPs are widely used in molecular imaging of cancer cells as probes for microscopy [16], antibacterial applications [17, 18], and so on. In this paper, we propose a simple and rapid method for Hg (II) ion using AgNPs.

## 2. Experimental

### 2.1. Reagents

Silver nitrate, trisodium citrate dihydrate, sodium borohydride, calcium chloride hexahydrate, cadmium chloride, and sodium chloride were purchased from Kelong Chemical Reagent Plant in Chengdu. Mercury (II) chloride, lead (II) nitrate, sodium nitrate, and magnesium chloride hexahydrate were purchased from Bo Yi, Chongqing Chemical Reagent Co., Ltd. Cupric sulfate and aluminum sulfate were obtained from Tianjin Damao Chemical Reagent Factory. Zinc chloride was obtained from Chongqing Inorganic Chemical Reagent Factory. All chemicals used in the experiment are analytical reagent grade without further purification. All water used is deionized water.

### 2.2. Apparatus

The absorption spectrum of AgNPs was measured on a TU-1901 UV-vis spectrophotometer (Beijing Purkinje General Instrument Company). The shape of AgNPs was imaged with a Hitachi S-4800 scanning electron microscopy (Tokyo, Japan). XW-80A vortex mixer purchased from Shanghai Jingke Industrial Co., Ltd was used to mix solutions. Reagents were exactly quantified by electronic balance, which was purchased from Sartorius Scientific Instrument (Beijing) Co., Ltd.

### 2.3. Preparation of AgNPs

AgNPs were prepared according to a previously published method [19]. 2 mL of 5% trisodium citrate solution was added to 100 mL solution of 0.5 mM AgNO_3_ under vigorously stirring. Then, 2 mL of sodium borohydride diluted solution was added into the above solution. AgNPs were formed rapidly and continuously stirred for 30 min. The AgNPs were placed overnight in a 4°C refrigerator.

### 2.4. General Procedures

In a 5 mL centrifugal tube, 300 *μ*L of HAc-NaAc buffer (pH 5.6), appropriate distilled water, different volume working solution of Hg (II) (2 × 10^−4^ mol/L), and 390 *μ*L AgNPs solution were added. The total volume of the mixed solution was 3 mL. After the solution was thoroughly mixed, absorbance spectrum was recorded by UV-vis spectrophotometer immediately.

## 3. Results and Discussion

### 3.1. Characteristics of Absorption Spectra

**Figure 1:**
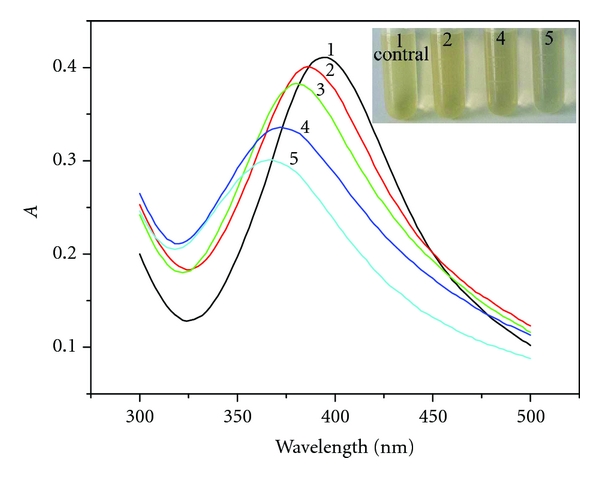
The absorption spectrum of AgNPs in the absence and presence of Hg (II) ion. (The inset picture displays color change corresponding to the curves: the solution color gradually changed from yellow to colorless.) Concentration: AgNPs: 6.5 × 10^−5^ mol/L; Hg (II) ion (from 1 curve to 5, ×10^−6^ mol/L): 0, 1.2, 3.2, 4.8, 6.0; HAc-NaAc buffer (pH 5.6).

AgNPs, as probe, are used to detect Hg (II) ion based on the blue shift of the maximum absorption wavelength. As shown in Figure 1, single AgNPs characterized at 394 nm (curve 1), when Hg (II) ion was added, the maximum absorption wavelength blue shifted, and the absorption intensity gradually decreased with increasing the Hg (II) ion content (curves 2–5). It is observed that the solution color gradually changed from yellow to colorless (the insert picture). Moreover, over the range from 2.0 × 10^−7^ to 6.0 × 10^−6 ^mol/L, the blue shift was in proportion to the Hg (II) concentration. Generally, when cations are adsorbed on the surface of AgNPs, the characteristic peak will red shift and broaden [20]. However, blue shift was observed in this experiment, which was induced by Hg (II) ion.

**Figure 2:**
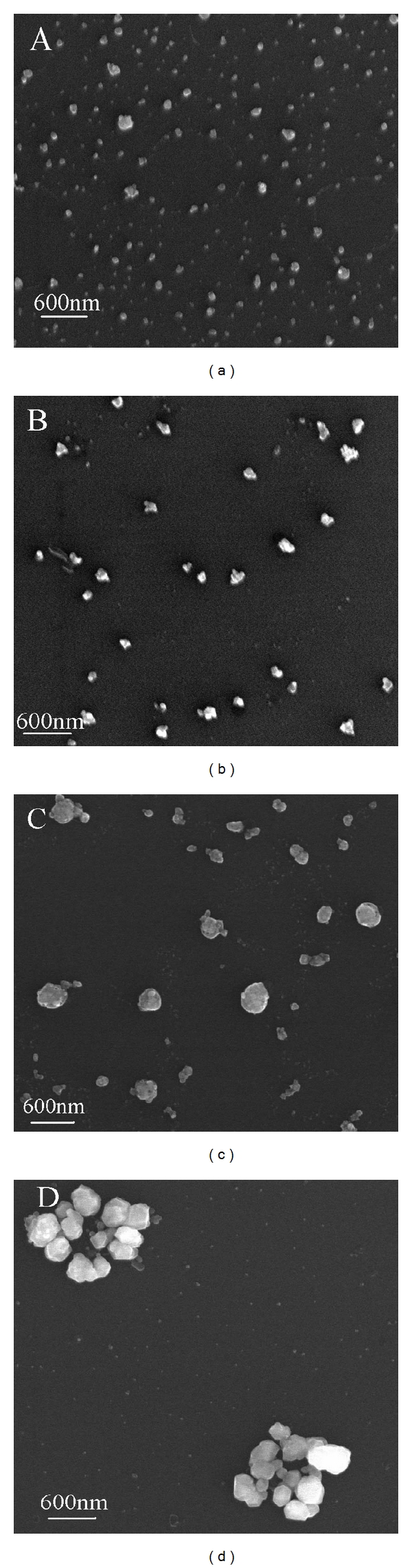
The scanning electron microscopy of AgNPs in the absence and presence of Hg (II) ion. Concentration: AgNPs: 6.5 × 10^−5^ mol/L; Hg (II) ion (from A to D, ×10^−6^ mol/L): 0, 2.0, 4.0, 6.0; HAc-NaAc buffer (pH 5.6).

In the preparation of AgNPs, sodium borohydride was reducing agent and in excess in order to keep AgNPs dispersed and stable. When Hg (II) ion was added into AgNPs solution, the in excess sodium borohydride could also reduce Hg (II) to Hg (0). Hg (0) atoms wrapped around silver nanoparticles and amalgam was generated, leading to the blue shift of the maximum peak [11, 20]. Up to now, articles about amalgam have been published. Amalgam could be formed between mercury and other metals, such as gold, silver, copper, tin, and zinc [21]. Silver or gold with mercury can form bimetallic colloids or the so-called amalgam and lead to blue shift of the absorption peak [11, 22]. In our experiment, the reaction of Hg (II) and AgNPs mixture resulted in the formation of silver amalgam and the blue shift. The images of scanning electron microscopy were carried out to demonstrate the above results. As shown in Figure 2, single AgNPs (Figure 2(a)) are dispersed well. However, the particle diameter gradually becomes larger when the Hg (II) ion concentration increases (Figures 2(b), 2(c), and 2(d)). It indicates that AgNPs are gradually wrapped and silver amalgam forms.

### 3.2. The Influence of Acidity

**Figure 3:**
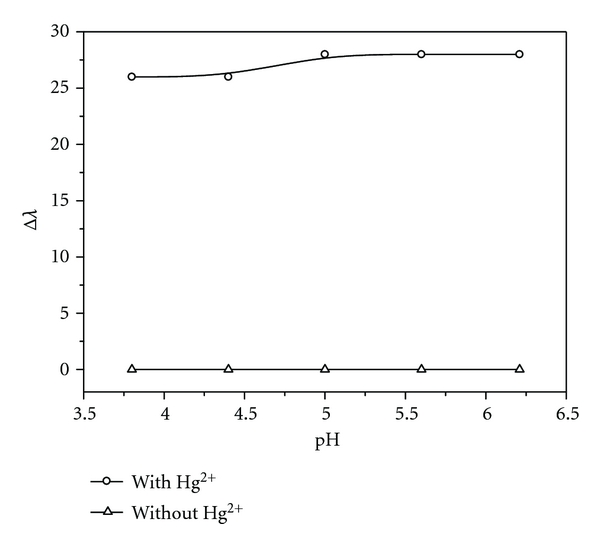
The influence of acidity. Concentration: AgNPs: 5.0 × 10^−5^ mol/L^−1^; Hg (II) ion: 6.0 × 10^−6^ mol/L; the pH of HAc-NaAc buffer (from 1 to 5): 3.8, 4.4, 5.0, 5.6, 6.2.

In order to determine the influence of pH on the system, the reaction was carried out at different pH value and the absorption was measured. According to the *K*sp of Hg(OH)_2_, we calculated that Hg (II) would deposit when pH was more than 6.24. So the acidity was controlled by HAc-NaAc buffer and keeping pH less than 6.24. Experimental results are shown in Figure 3. It is displayed that the acidity has slight influence on the experimental results. When the pH was near weak acidity, the blue-shifted maximum absorption wavelength was a little larger than that near the strong acidity. Finally, pH 5.6 was chosen to be our experimental condition.

### 3.3. The Influence of the Concentration of AgNPs

**Figure 4:**
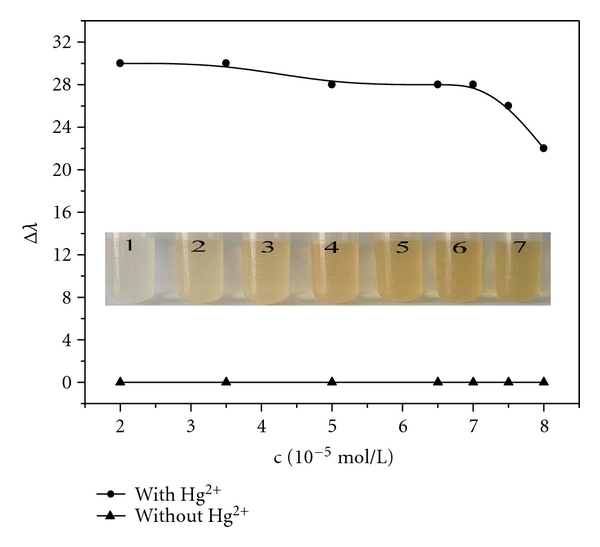
Effect of the AgNPs concentration. Concentration: AgNPs (×10^−5^ mol/L): 2.0, 3.5, 5.0, 6.5, 7.0, 7.5, 8.0, respectively; Hg (II) ion: 6.0 × 10^−6^ mol/L; HAc-NaAc buffer (pH 5.6). The inset picture displays color change with different concentration of AgNPs.

To select the optimal AgNPs concentration in our experimental system, we also studied different concentration of AgNPs. The results can be seen in Figure 4. First, when the concentration was over the range from 2.0 × 10^−5^ mol/L to 7.0 × 10^−5^ mol/L, the blue shift of the maximum absorption wavelength is nearly the same. When its concentration is larger than 7.0 × 10^−5^ mol/L, the blue shift is sharply decreased. Second, the color was stronger with increasing the AgNPs concentration. Considering the two factors, we choose 6.5 × 10^−5^ mol/L as the optimal concentration of AgNPs.

### 3.4. Selectivity and Stability of the Reactive System

**Figure 5:**
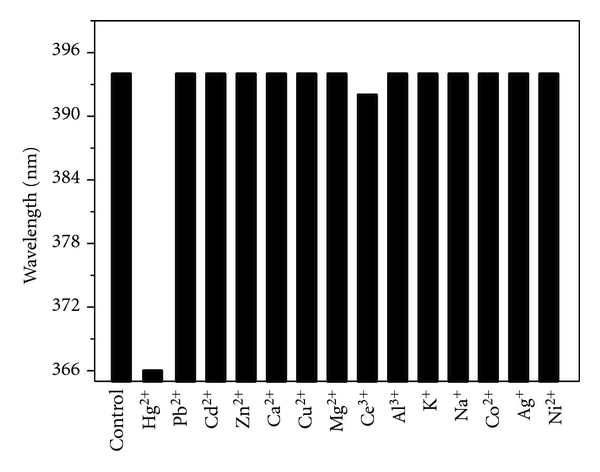
Selectivity of the reactive system. Concentration: AgNPs: 6.5 × 10^−5^ mol/L; Hg (II) ion: 6.0 × 10^−6^ mol/L; the other metal ions: 6.7 × 10^−6^ mol/L; HAc-NaAc buffer (pH 5.6).

**Figure 6:**
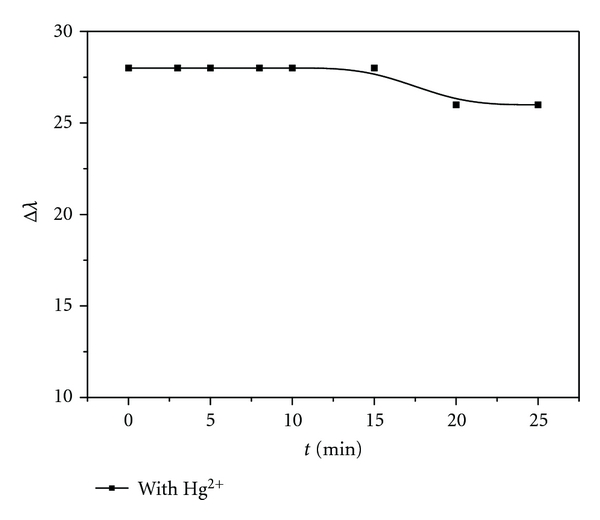
Stability of the reactive system. Concentration: AgNPs: 6.5 × 10^−5^ mol/L; Hg (II) ion: 6.0 × 10^−6^ mol/L; *t* (min): 0, 3, 5, 8, 10, 15, 20, 25, respectively; HAc-NaAc buffer (pH 5.6).

We also studied the selectivity and stability of the system. The experimental data are shown in Figures 5 and 6.  Figure 5 indicates that the reactive system has a very good selectivity on Hg (II) ion. When Hg (II) ion is added, the maximum absorption wavelength of AgNPs significantly changed. While other metal ions are added, respectively, including Pb^2+^, Ni^2+^, Cd^2+^, Zn^2+^, Ca^2+^, Cu^2+^, Mg^2+^, Ce^3+^, Al^3+^, K^+^, Ag^+^, Na^+^, Co^2+^, the characteristic peak still stay at 394 nm, except Ce^3+^, which only induced a weak blue shift of 2 nm.

In addition, chloride (Cl^−^) caused great interference on our experiment, which could be eliminated easily by Ag_2_O [23]. So a simple pretreatment was needed to screen Cl^−^ before determination.

We investigated the stability of the reactive system. It could be stable for at least 15 min, as shown in Figure 6. In the presence of Hg (II) ion, silver amalgam was formed quickly, and the particle diameter increased. It was easy to deposit down for larger particles. Therefore, the reactive system could not be stable for long time. The images of scanning electron microscopy also indicated our explanation (seen in Figure 2).

### 3.5. Determination of Hg (II) Ion

**Figure 7:**
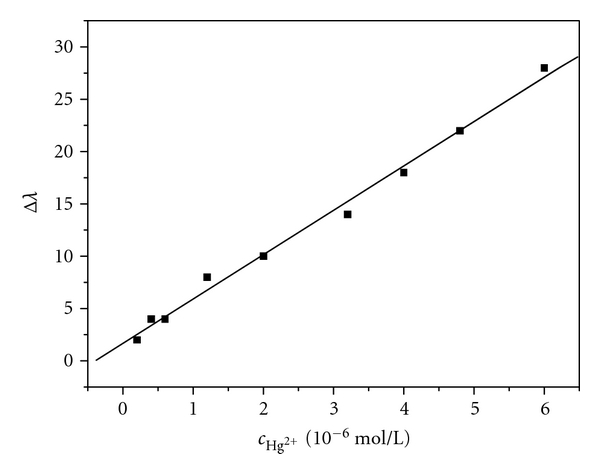
The blue shift of the maximum absorption wavelength of AgNPs and Hg (II) ion concentration. A linear correlation exists over the range of 2.0 × 10^−7^–6.0 × 10^−6^ mol/L.

Under the optimal conditions, the calibration curve for detecting Hg (II) ion was established. There was a good linear correlation between the blue shift of maximum absorption wavelength and the concentration of Hg (II) ion over the range of 2.0 × 10^−7 ^mol/L to 6.0 × 10^−6 ^mol/L, with the limit of detection (3**σ**) of 6.6 × 10^−9 ^mol/L. And its standard regression equation was Δ*λ* = 1.6708 + 4.23943*c*_Hg^2+^_(*r* = 0.9961, *n* = 9), as shown in Figure 7.

According to the calibration curve, three synthetic samples of Hg (II) ion containing some other metal ions were detected. The measurement results are listed in Table 1. It is shown that the recovery is between 83.5% and 110.0%, and the R.S.D is lower than 11.2%.

## 4. Summary

Hg (II) ion can interact with AgNPs solution prepared by sodium borohydride. In the presence of Hg (II) ion, the AgNPs solution displays color change and blue shift of the maximum absorption wavelength, and thus we propose a rapid, simple, and highly selective method for detecting Hg (II) ion. The influence of acidity and the concentration of AgNPs, selectivity, and stability are all studied for the reactive system. Here, we expect that it will become a promising technique to determine Hg (II) ion.

## Figures and Tables

**Table 1:** The results of synthetic samples.

Samples	Main interferes	Found (*μ*mol/L)	Recovery (%)	R.S.D. (%) (*n* = 5)
1	K^+^, Cd^2+^, Ca^2+^, Al^3+^.	1.73–2.20	86.4–110.0	9.8
2	Cu^2+^, Mg^2+^, Pb^2+^, Na^+^.	2.67–3.38	83.5–105.6	11.2
3	Zn^2+^, Ce^3+^, Ag^+^, Co^2+^, Ni^2+^.	4.32–4.56	90.0–95.0	3.0

AgNPs: 6.5 × 10^−5^ mol/L; other metal ions: 6.7 × 10^−6^ mol/L; HAc-NaAc buffer (pH 5.6).
